# OTU deubiquitinase 4 is silenced and radiosensitizes non-small cell lung cancer cells via inhibiting DNA repair

**DOI:** 10.1186/s12935-019-0816-z

**Published:** 2019-04-15

**Authors:** Zhiqiang Wu, Minghan Qiu, Yu Guo, Jinlin Zhao, Zhuang Liu, Hui Wang, Maobin Meng, Zhiyong Yuan, Zeyun Mi

**Affiliations:** 1 Department of Radiation Oncology, Tianjin Medical University Cancer Institute & Hospital, Key Laboratory of Cancer Prevention and Therapy, National Clinical Research Center for Cancer, Tianjin’s Clinical Research Center for Cancer, Tianjin, 300060 China; 2 Department of General Surgery, The First Affiliated Hospital, Sun Yat-sen University, Guangzhou, Guangdong China; 3 Department of Biochemistry and Molecular Biology, College of Basic Medical Science, Tianjin Medical University, Tianjin, 300070 China

**Keywords:** Non-small cell lung cancer, Radiosensitizer, OTUD4, Homology repair, DNA methylation

## Abstract

**Background:**

Radiotherapy is becoming one major therapeutics for non-small cell lung cancer (NSCLC). Identifying novel radiosensitizers will greatly increase the efficacy of radiotherapy and benefit more patients. OTU deubiquitinase 4 (OTUD4) has been reported involved in DNA damage repair pathways and could be a potential target for chemotherapy therapy. This study aimed to investigate the roles of OTUD4 in regulation of radiosensitivity of NSCLC via modulating DNA repair.

**Methods:**

The expression of OTUD4, γ-H2Ax and ATM/CHK2/p53 pathway-related signaling molecules were detected by Western blotting and QRT-PCR. The methylation of OTUD4 promoter was investigated by 5-aza-deoxycytidine treatment, methylation-specific PCR and bisulfite genomic sequencing assays. Radiosensitivity was assessed by the clonogenic formation assay. Cell cycle, cell apoptosis were analyzed by flow cytometry. DNA damage and repair were determined by comet assay, γ-H2Ax foci staining and flow cytometry.

**Results:**

OTUD4 is dramatically downregulated in NSCLC and its downregulation significantly correlates with poor prognosis of NSCLC patients. Promoter hypermethylation is responsible for the loss of OTUD4 expression in NSCLC cells. Overexpression of OTUD4 increases radiosensitivity of NSCLC cells exhibiting as impaired clonogenic formation ability, enhanced cell cycle arrest and increased cell apoptosis. Moreover, molecular mechanism study reveals that OTUD4 radiosensitizs NSCLC cells via ATM/CHK2/P53 signaling and inhibiting homology-directed repair of DNA double strand breaks induced by ionizing radiation.

**Conclusions:**

This study uncovers a tumor-suppressing role of OTUD4 and that OTUD4 is a potential radiosensitizer for NSCLC.

**Electronic supplementary material:**

The online version of this article (10.1186/s12935-019-0816-z) contains supplementary material, which is available to authorized users.

## Background

Lung cancer is the most frequently diagnosed cancer and the leading cause of cancer related death, with an estimated over 1.8 million new cases and 1.5 million deaths world widely in 2012 [1]. Non-small cell lung cancer (NSCLC) is the main histological type of lung cancer, which accounts for nearly 85% of all cases of lung cancer [2]. Thus, it is a big challenge to fight against NSCLC. Radiotherapy is becoming more and more important for treatment of NSCLC. Recent years, radiotherapy, especially stereotactic body radiotherapy (SBRT), achieved promising benefit for NSCLC patients [3]. However, radioresistance hinders further improving efficacy of radiotherapy and results in treatment failure in patients. Therefore, elucidating the mechanism of radioresistance and finding/developing effective radiosensitizer are very important.

The primary effect of ionizing radiation (IR) is bringing damages to cellular DNA directly and indirectly [4]. Upon DNA damage, ATM is quickly recruited to the DSBs sites in an MRE11-RAD50-NBS1 (MRN) complex dependent way, and subsequently activated via autophosphorylation [5]. The histone variant H2AX is phosphorylated by activated ATM at serine 139 residue. Phosphorylated H2AX (termed γ-H2AX) then incorporates into nucleosomes instead of the core histone at the DNA damage sites, which can spread about 2 megabases of chromatin region or thousands of nucleosomes and formed nuclear foci when stained with specific antibody [6]. γ-H2AX recognizing antibodies have become a gold standard for detecting the presence of DSBs [7, 8]. In addition to H2AX, activated ATM phosphorylates lots of other proteins involved in DNA damage response (DDR) and repair signaling pathways, such as cell cycle check point, DNA repair, apoptosis, and so forth.

DNA double strand breaks (DSBs) are considered the most cytotoxic type of DNA lesion induced by IR. Homologous recombination (HR) repair is important for DSBs precise repair. Modulating factors that are essential for HR affects sensitivity to ionizing radiation [9, 10]. OTUD4 has been reported to take part in DNA damage repair pathways including GG-NER and alkylation damage repair pathway [11, 12]. Moreover, knockdown of OTUD4 significantly increased the sensitivity of PC-3 and H23 tumor cells to methyl methanesulfonate, an alkylating agent which leads to DNA alkylation damage [12]. Hence, OTUD4 could be a potential target for cancer therapy via regulating DNA damage repair. However, whether OTUD4 participates in DSBs repair, especially HR, and modulates radiosensitivity of cancer cells remain to be elucidated.

In this study, we find that OTUD4 is significantly downregulated in NSCLC cell lines and patient specimens. Promoter methylation accounts for the depression of OTUD4 in NSCLC. Decreased expression of OTUD4 significantly correlates with poor prognosis of NSCLC patients. Moreover, overexpression of OTUD4 could increase radiosensitivity as evidenced by impaired clonogenic formation ability, increased cell cycle arrest and enhanced cell death after ionizing radiation treatment. Further investigation proved that OTUD4 inhibit DSBs homology repair (HR). Together, this study implicates that OTUD4 is a potential radiosensitizer for NSCLC.

## Materials and methods

### Patient tissue specimens

Patient consent and approval from the Institutional Research Ethics Committee of Tianjin Cancer Institute and Hospital were obtained for the use of the clinical materials for research purposes. Ten primary NSCLC tumor and adjacent non-tumor tissue sample pairs were obtained from the Department of Pathology, Tianjin Medical University Cancer Hospital. Fresh tumor tissues were snap-frozen in liquid nitrogen in the operating room and then stored at − 80 °C. The tumor tissues were histopathologically and clinically diagnosed using both fresh and formalin-fixed, paraffin-embedded tumor tissues. All fresh samples were confirmed by hematoxylin and eosin (H&E) staining in frozen sections for the histopathological analysis.

### Cell culture

Non-small cell lung cancer cell lines, including A549, H292, H460, H520, H1299, H1975 and PC-9, were cultured in RPMI 1640 medium (Gibco, Rockville, MD, USA). 293FT cells were cultured in DMEM medium (Gibco). DR-GFP-U2OS and EJ5-U2OS cell line [13, 14] were shared by Prof. Lei Shi’s laboratory at Tianjin Medical University and cultured in DMEM medium. The medium were supplemented with 10% FBS, 1% Non-essential amino acids, 2 mM l-glutamine, 100 U/ml penicillin, and 100 μg/ml streptomycin (Gibco). Lung bronchus epithelial cell line BEAS-2B was cultured in BEGM medium (Lonza).

### Plasmids, transfection and stable cell line construction

OTUD4 was PCR amplified from cDNA of BEAS-2B cells with C-terminus flag-tagged and subcloned into the BamH I/SpeI sites of pSin-EF2-puro vector and validated by sequencing. Plasmids pSin-EF2-OTUD4, psPAX2 and pMD2.G were cotransfected into 293FT cells with Lipofectamine-2000 (Invitrogen) for virus package. Twenty-four and 48 h later, the virus-containing medium were collected, filtered and infected H460 and A549 cells daily. The infected cells were then selected with medium containing puromycin (1 μg/ml, Sigma) for at least 1 week and further maintained at medium containing low concentration of puromycin (0.5 μg/ml).

### Western blotting and antibodies

Western blotting was conducted as previously reported [15]. The following antibodies were used: OTUD4 (#ABN477) was form Millipore; γ-H2AX (#9718), p-CHK2 (#2661), p53 (#2524), p21 (#2947), p-ATM (#5883) and Caspase-3 (#9662) were form Cell Signaling Technology; ATM (A1106) and α-tubulin (T9026) were from Sigma-Aldrich; CHK2 (sc-17747) and GAPDH (sc-365062) were from Santa Cruz Biotechnology.

### RNA extraction, reverse transcription (RT) and real-time PCR

Total RNA was extracted using Trizol reagent (Invitrogen). cDNA was synthesized using M-MLV Reverse Transcriptase (Promega). FastStart Universal SYBR Green Master (Roche) was used for quantitative real-time PCR. Amplification and real-time analysis was performed with BioRad CFX96 system (BioRad). Primers were as following: OTUD4, forward 5′-AGACCCGAACCAAGCACAT-3′, and reverse 5′-CTGGCTTTTGTTCCGCA-3′; GAPDH, forward 5′-GAAGGTGAAGGTCGGAGT CA-3′, and reverse 5′-TTGAGGTCAATGAAGGGGTC-3′. Transcript levels were normalized to the housekeeping gene GAPDH levels. The relative mRNA levels were calculated according to the comparative Ct (ΔΔCt) method, where Ct represents the threshold cycle for each transcript.

### Methylation specific PCR

Methylation specific PCR (MSP) was performed according to a previous study [16]. Briefly, Genomic DNA was extracted using TIANamp Genomic DNA Kit (TIANGEN). 2 μg of genomic DNA was bisulfite-treated with EpiTect Bisulfite Kit (Qiagen). Bisulphite-treated DNA was amplified using primers specific for either methylated or unmethylated DNA. The sequences of methylated-specific (M) primer and unmethylated-specific (U) primer for OTUD4 were: OTUD4 (M), forward 5′-CGTTACGGTTCGGAATAGGTAC-3′, reverse 5′-ACATAAAAACTACCGTCGA CGTC-3′; OTUD4 (U), forward 5′-TTGTTATGGTTTGGAATAGGTATGA-3′, reverse 5′-CAACATAAAAACTACCATCAACATC-3′.

### Bisulfite genomic sequencing

Genomic DNA from different cells and patients’ tumor tissues were bisulfite-treated with the Epitect Bisulfite Kit (Qiagen). Bisulfite-treated DNA was amplified with bisulfite-sequencing PCR (BSP) primers located in the OTUD4 promoter. The primers sequences were: forward, 5′-AGGGTTAGTTTTATATGGTTAGGT-3′, reverse, 5′-ACCTAAATCCTAAATCAAACAAC-3′. PCR products were purified and ligated into pMD19-T Vector System (Takara). Plasmids from single colonies were extracted and sequenced. At least 3 clones of each sample were selected for sequencing.

### Cell clonogenic formation assay

The cell clonogenic formation assay was performed according to a previous report [17]. Briefly, cells were trypsinized and suspended. Then 200, 300, 1000, 5000, or 10000 H460 cells or 200, 300, 400, 1000 and 4000 A549 cells were seeded into 6-well cell culture plates in triplicates respectively. After attaching to the plates (6 to 8 h), the cells were treated with single-dose irradiation (0, 2, 4, 6, or 8 Gy). After incubated for about 2 weeks, cultures were fixed with pechiled methanol and stained with 0.1% crystal violet. The number of colonies with > 50 cells was counted under a dissecting microscope. The percentage of cell survival was calculated. The experiment conducted at least three times.

### Cell cycle analysis

Cells were irradiated with 6 Gy dosage and allowed to recover for 12 h. Cells were fixed, stained with PI, and analyzed by flow cytometry as previous reported [18]. Thirty thousand cells were analyzed by using a FACS alibur instrument (BD Biosciences). The cell cycle distribution was assessed using ModFit LT 3.1 trial cell cycle analysis software. Representative images from three independent experiments are shown.

### Single-cell gel electrophoresis (comet) assay

Cells were irradiated and allowed to recover for 3 h prior to analysis. The single cell comet assays were carried using CometAssay Kit (4250-050-K, Trevigen) following the manufacturer’s instructions. DNA damage was quantified for at least 50 cells for each experimental condition by determining percent of DNA in the tail relative to the total DNA using the software Open Comet_ImageJ.

### Immunofluorescence microscopy

Cells were trypsinized and seeded on coverslips (Thermo Fisher Scientific, Lafayette, CO) in 24-well plate overnight. The next day cells were irradiated with single dose and allowed to recover for indicated time prior to analysis. Immunostaining was performed according to a previous report [15]. Anti-γH2AX (#9718, 1:200; Cell signaling Technology), anti-Rad51 (ab63801, Abcam) and species-specific FITC-conjugated secondary antibodies (1:500; Invitrogen) were used. Gray level images were acquired under a laser scanning microscope (Axio Imager.Z2, Carl Zeiss Co. Ltd.). Images were captured using the AxioVision Rel.4.6 computerized image analysis system. At least 50 cells were counted for quantification of γ-H2AX foci.

### Homologous recombination reporter assay

4 × 10^5 DR-GFP-U2OS or EJ5-U2OS cells were seeded into 6-cm plates. Next day, 2 μg of pCMV-I-SceI [19] and 2 μg of pSin-EF2-OTUD4 or vector plasmid were cotransfected into cells using Lipofectamin-2000 (Invitrogen). Forty-eight hours later, GFP-positive cells was quantitated by flow cytometric analysis (FACS Calibur; BD Biosciences). Representative images from three independent experiments are shown.

### Statistical analysis

Statistical analysis was performed using GraphPad Prism 5 (GraphPad Softare Inc.). Unless indicated otherwise, all results were presented as mean ± SD. Two-tailed, unpaired student’s t test was used for comparisons between groups for statistical significance. p < 0.05 in all cases was considered statistically significant.

## Results

### OTUD4 is downregulated in lung cancer and its downregulation associated with poor prognosis of patients with lung cancer

**Fig. 1:**
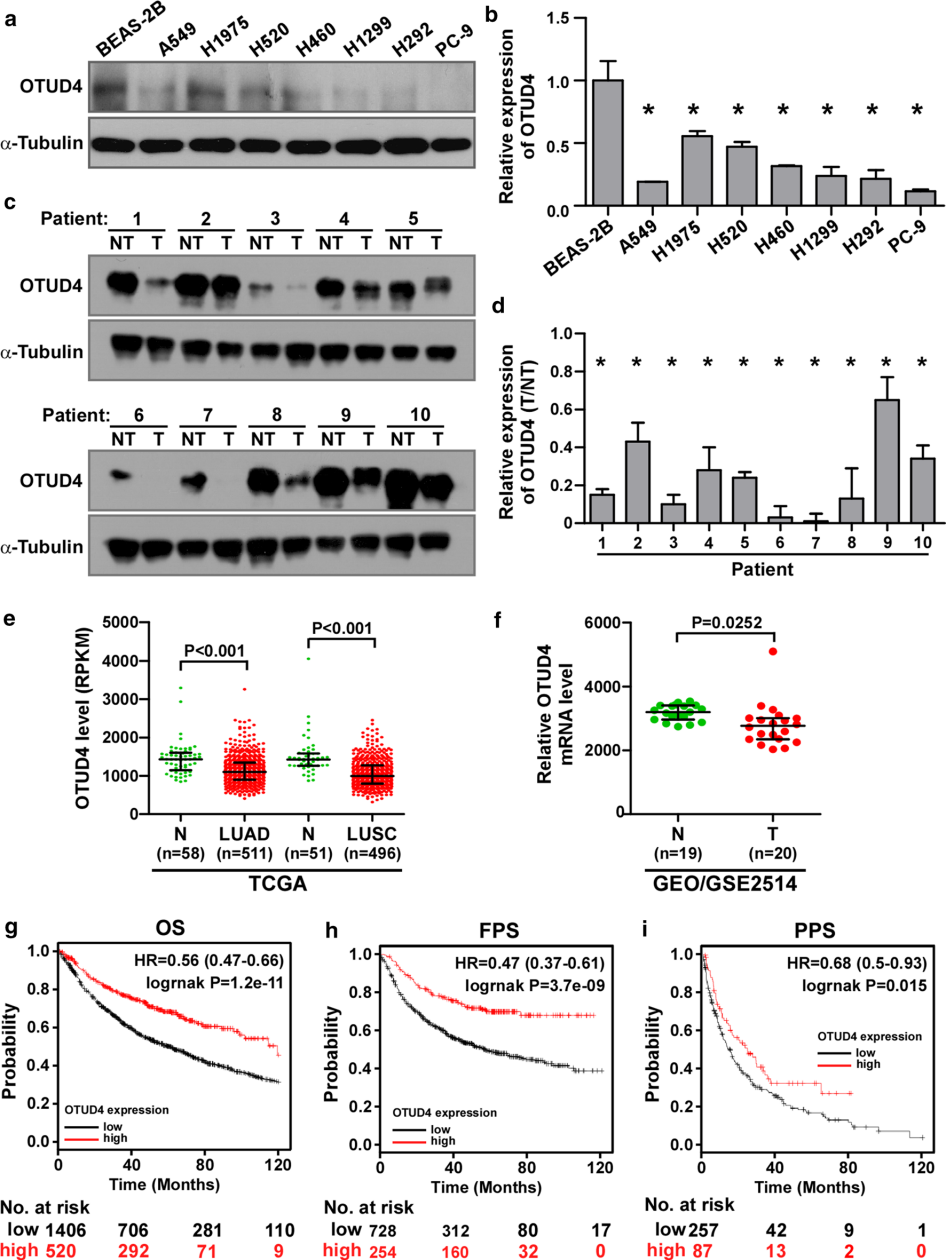
OTUD4 is downregulated and associates with poor prognosis in NSCLC. Western blotting (**a**) and real-time PCR (**b**) analysis of the expression of OTUD4 in NSCLC cell lines. Western blotting (**c**) and real-time PCR (**d**) analysis of the expression of OTUD4 in paired adjacent non-tumor (NT) and tumor (T) tissues from NSCLC patients. **e** Expression level of OTUD4 in normal and tumor tissues of patients from the TCGA dataset. Error bars represent Median with interquartile range. N, normal. LUAD, lung adenocarcinoma. LUSC, lung squamous cell carcinoma. **f** Expression level of OTUD4 in normal (N) and tumor (T) tissues of patients from the GEO/GSE2514 dataset. Error bars represent Median with interquartile range. Kaplan–Meier analysis of the OS (**g**), FPS (**h**) and PPS (**i**) in lung cancer using Kaplan–Meier Plotter (http://kmplot.com/analysis/). OS, overall survival. FP, first progression. PPS, post progression survival. Tubulin served as loading control. *p < 0.05

First, the expression of OTUD4 in non-small cell lung cancer was analyzed. OTUD4 was dramatically downregulated in NSCLC cell lines (Fig. 1a, b) and tumor tissues (Fig. 1c, d) compared with the lung bronchus epithelial cell BEAS-2B cells and the matched adjacent non-tumor tissues respectively in both protein and RNA level. Analysis of TCGA dataset showed that OTUD4 level was significantly depressed in both lung adenocarcinoma (LUAD) and squamous cell carcinoma (LUSC) tissues compared with normal lung tissues (Fig. 1e). Moreover, data form published lung cancer patient gene expression profiles (NCBI/GEO/GSE2514, n = 39) also indicated decreased expression of OTUD4 in tumor tissues. What’s more, assessment using the Kaplan–Meier Plotter (http://kmplot.com/analysis/index.php?p=service&cancer=lung) showed that downregulated OTUD4 significantly correlated with poorer overall survival (OS, HR = 0.56, logrank P = 1.2e−11), first progression survival (FPS, HR = 0.47, logrank P = 3.7e−9) and post progression survival (PPS, HR = 0.68, logrank P = 0.015) (Fig. 1g–i) in lung cancer. These data suggest that OTUD4 is tumor-suppressing and a novel prognostic biomarker for lung cancer.

### Hypermethylation in the promoter region accounts for depression of OTUD4

**Fig. 2:**
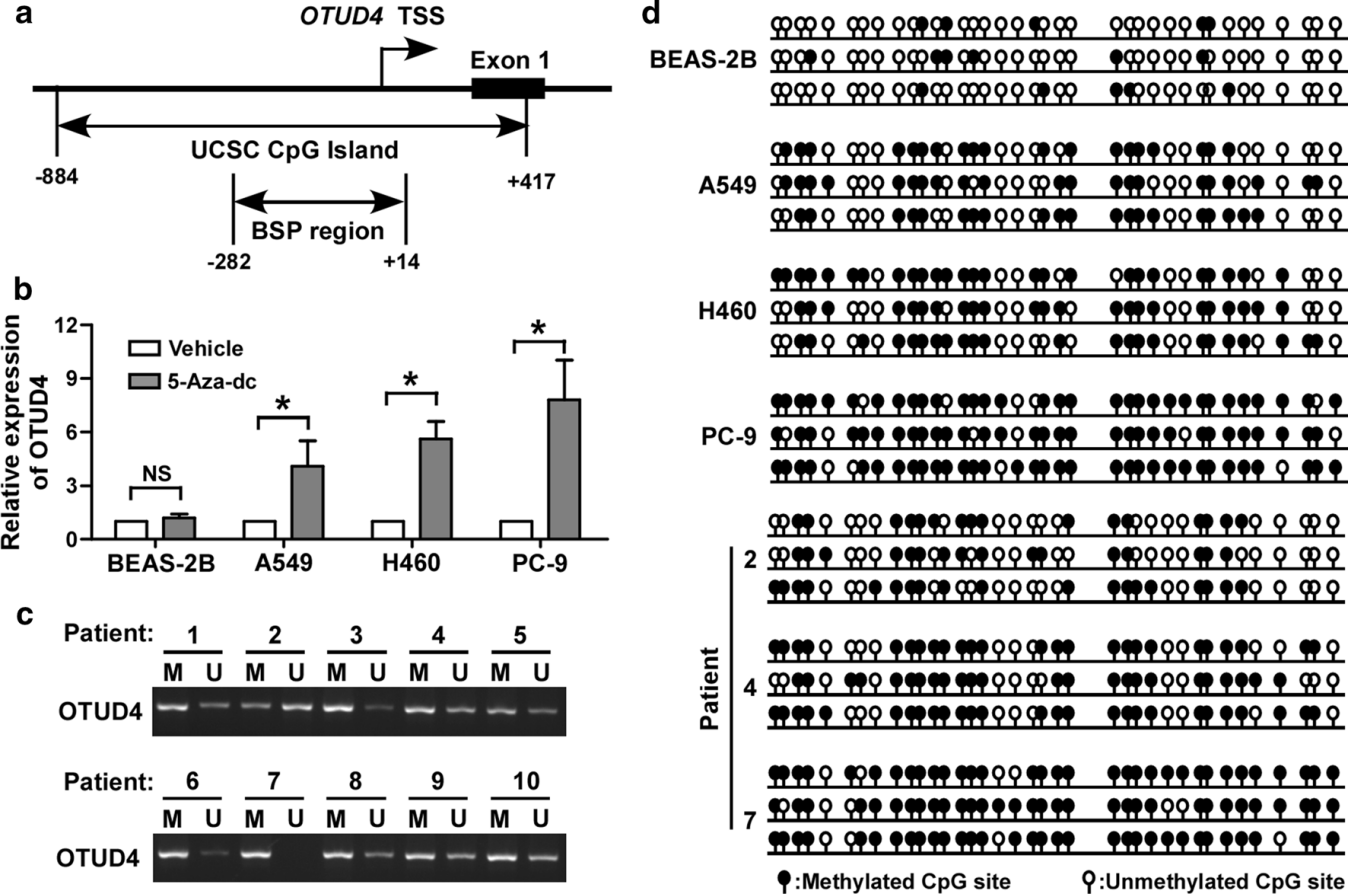
Promoter methylation is responsible for the loss of OTUD4 expression in NSCLC cells. **a** Schematic illustration of the arrangement of the promoter region and the region for BSP analysis. **b** Expression of OTUD4 mRNA in indicated cells treated with or without 5-Aza-dc. **c** Methylation specific PCR analysis of genomic DNA of tumor tissues from 10 patients. **d** Methylation status of promoter CpG islands from 3 individual clones analyzed by BSP. Error bars represent SD from 3 independent experiments. *p < 0.05. BSP, bisulfite genomic sequencing

OTUD4 is downregulated of in NSCLC, then we explored the underlying mechanism. Epigenetic modifications, such as DNA methylation and histone modification, play pivotal roles in regulating gene expression. A CpG islands rich region of human OTUD4 promoter was retrieved from the UCSC genome browser (http://genome.ucsc.edu/, Fig. 2a), which suggested that promoter methylation might repressed OTUD4 expression. 5-Aza-dc, a DNA methyltransferase inhibitor, significantly promoted the expression of OTUD4, especially in cell lines with low levels of OTUD4, but not in the BEAS-2B cells (Fig. 2b). MSP assay showed that OTUD4 promoter genomic DNA was largely methylated in tumor tissues form 10 NSCLC patients (Fig. 2c). Moreover, BSP result showed that in these analyzed NSCLC cell lines and tissues the CpG islands in the OTUD4 promoter were hypermethylated (Fig. 2d). These data strongly suggested an essential role of promoter methylation in OTUD4 depression in NSCLC.

### OTUD4 radiosensitizes NSCLC cells

**Fig. 3:**
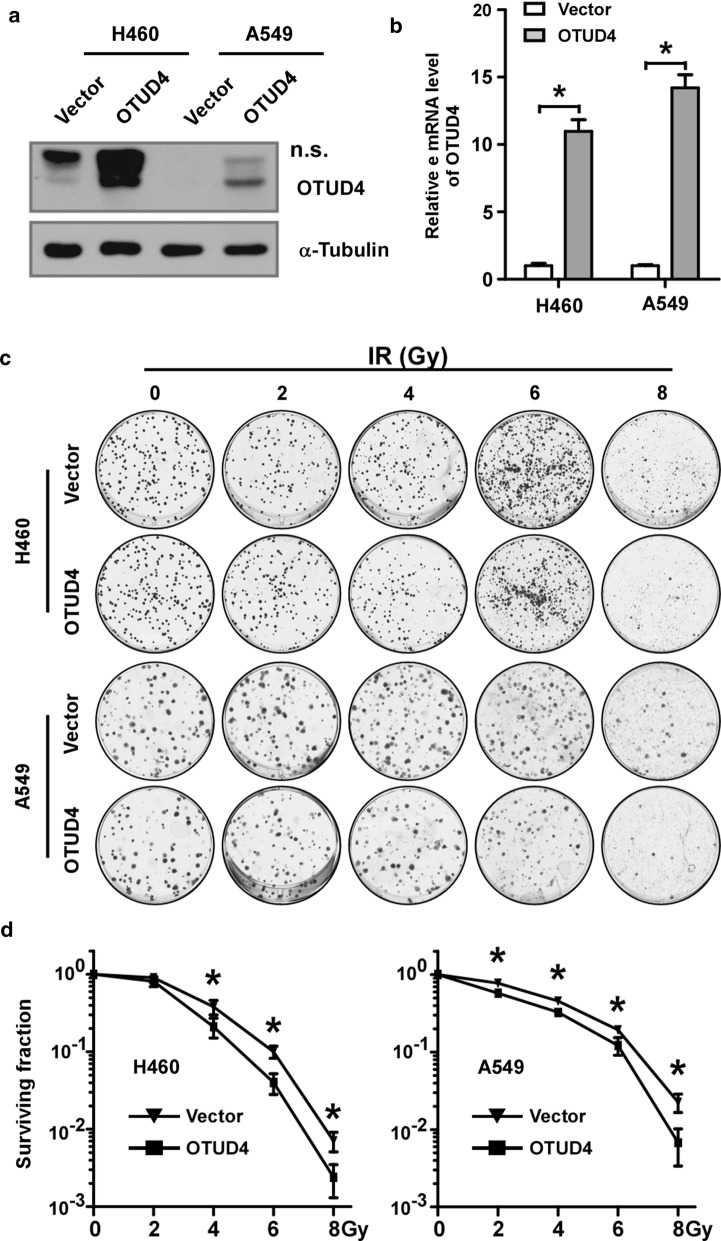
Overexpression of OTUD4 increases radiosensitivity of NSCLC cells. Western blotting (**a**) and real-time PCR (**b**) validating overexpression of OTUD4 in indicated cells. **c** Representative images of cell clonogenic formation. **d** Statistical quantification of cell clonogenic formation of indicated cells treated with different dosage of IR. α-Tubulin served as loading control. Error bars represent SD from 3 independent experiments. n.s., non-specific. *p < 0.05

Radiotherapy kills tumor cells by inducing lethal DNA damage and is one of the major therapeutics for lung cancer. It has been reported that OTUD4 involved in DNA repair [11, 12], thus we hypothesized that OTUD4 played a role in regulating radiosensitivity of NSCLC. Because OTUD4 was dramatically downregulated and hardly detected in NSCLC cell lines (Fig. 1a), we overexpressed OTUD4 in H460, A549 and PC-9 cells (Fig. 3a, b and Additional file 1: Fig. S1A). Clonogenic formation assay showed that overexpression of OTUD4 significantly decreased colonies formation efficiency after IR treatment (Fig. 3c, d and Additional file 1: Fig. S1B, C), which means that overexpression of OTUD4 did increase radiosensitivity of lung cancer cells. These data proved that OTUD4 could be a radiosensitizer of NSCLC.

### Overexpression of OTUD4 promotes G2/M cell cycle arrest and apoptosis

**Fig. 4:**
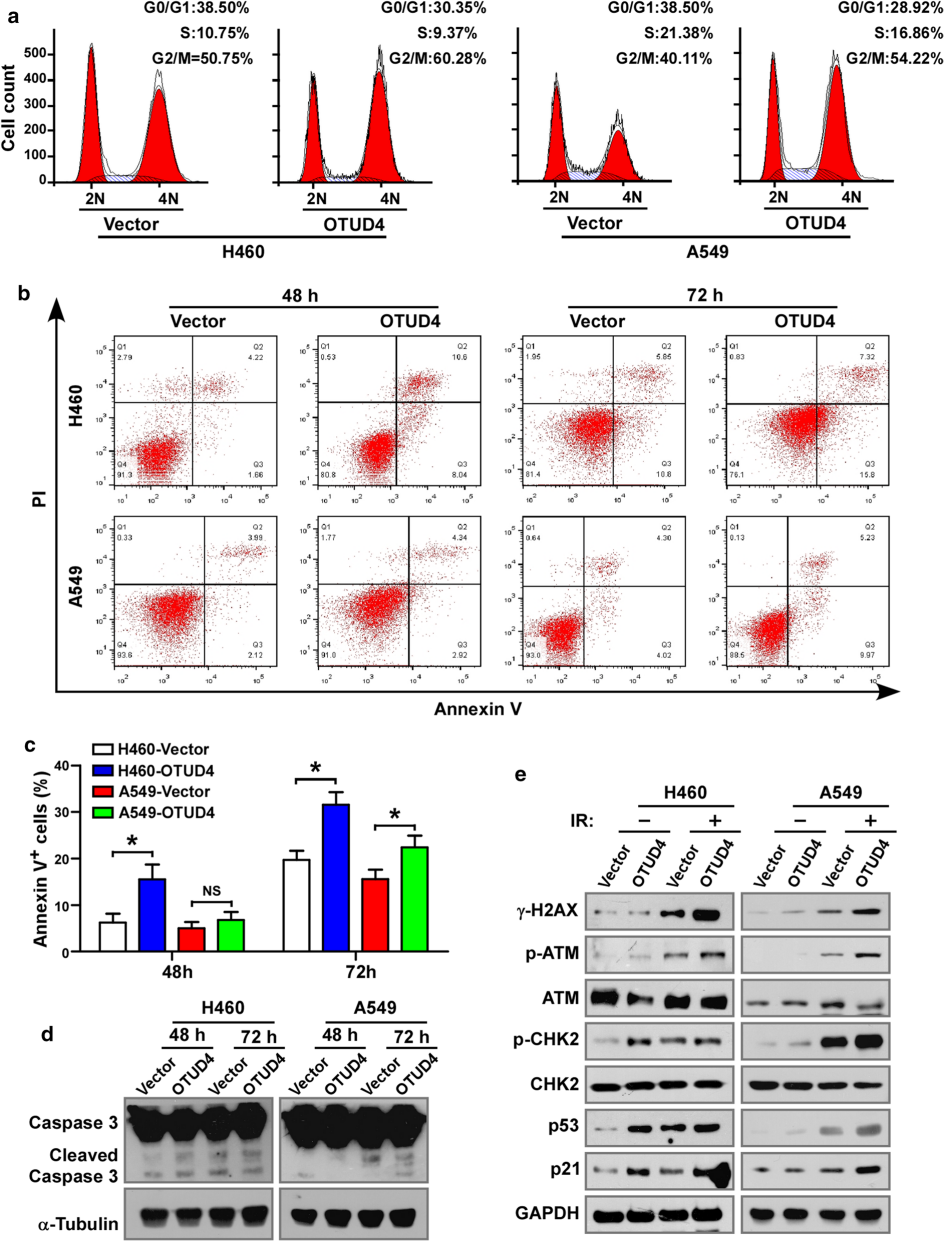
OTUD4 enhances cell cycle arrest and promotes cell apoptosis induced by IR. **a** Representative pictures of cell cycle distribution of indicated cells 12 h after irradiation. Representative images (**b**) and statistical quantification (**c**) of irradiation induced cell death analyzed by FACS. **d** Western blotting analysis of the expression of Caspase-3 and cleaved Caspase-3 in irradiated cells. **e** Western blotting analysis p-ATM, total ATM, p-CHK2, total CHK2, p53, p21 and γ-H2AX in indicated cells treated with or without irradiation. α-Tubulin and GAPDH served as loading control. Error bars represent SD from 3 independent experiments. *p < 0.05

Next, we analyzed whether OTUD4 regulates the DNA damage responses to IR. Cell cycle analysis showed that overexpression of OTUD4 slightly increased cell percentage in G0/G1 phase (Additional file 2: Fig. S2A). Moreover, the proportion of cells with G2/M phase arrest was significantly increased in A549 and H446 cells with OTUD4 overexpression compared to vector control 12 h after irradiated (Fig. 4a). Additionally, upregulation of OTUD4 promoted cell death induced by IR, indicating by increased percentage of annexin V positive cells (Fig. 4b, c) and enhanced expression of active Caspase 3 (Cleaved Caspase 3, Fig. 4d), but not the untreated cells (Additional file 2: Fig. S2B and C). What’s more, increased expression of phosphorylated H2AX, ATM and CHK2 were noticed in OTUD4 overexpressed cells (Fig. 4e). P53 and its downstream gene p21, two major players in the apoptosis and cell cycle arrest responding to DNA damage, were also upregulated in OTUD4 overexpressed cells (Fig. 4e). Together, these findings revealed that OTUD4 enhanced G2/M arrest and apoptosis induced by IR in NSCLC cells.

### OTUD4 inhibits homology-directed DNA repair

**Fig. 5:**
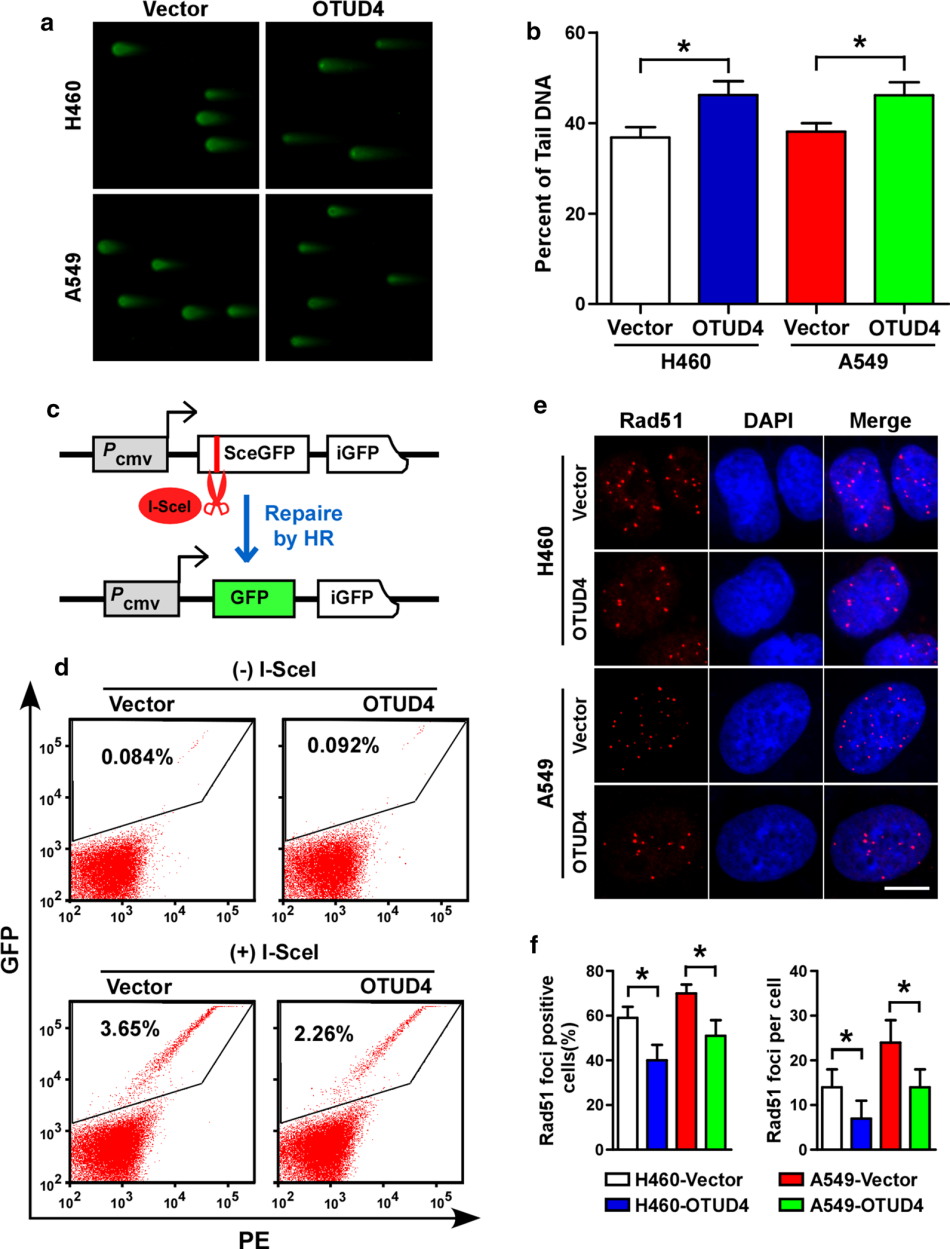
OTUD4 inhibits DNA damage repair. Representative pictures (**a**) and quantification (**b**) of unrepaired DSB analyzed by comet assay 3 h after irradiation. Diagram (**c**) and homology repair efficiency (**d**) determined by FACS of DR-GFP-U2OS cells transfected with indicated plasmid. Representative pictures (**e**) and quantification (**f**) of Rad51 foci in vector and OTUD4 overexpressed cells treated with IR (6 Gy) and allowed recovering for 12 h. Scale bar, 10 μm. Error bars represent SD from 3 independent experiments. *p < 0.05

DNA damage, especially DSBs, is the major cause of cell death induced by IR. Comet assay showed that A549 and H460 cells with OTUD4 overexpressed had significantly higher residual DNA damage relative to control cells (Fig. 5a, b). γ-H2AX foci, indicating DNA lesions, persist longer in radiosensitive cells than in radioresistant cells upon irradiation [20, 21]. After IR treatment, γ-H2AX foci were significantly increased and persisted longer in OTUD4 overexpressed cells than vector control cells (Additional file 3: Fig. S3A-B). Theas results suggested that OTUD4 impaired DNA repair efficiency of NSCLC cells.

DSBs is the main cause of cell death induced by IR. HR and non-homologous end joining (NHEJ) are the two major ways to repair DSBs. Next, the effect of OTUD4 on HR and NHEJ DNA repair signaling was evaluated using DR-GFP U2OS and EJ5-U2OS reporter cells respectively [13, 14]. With these reporters, DSBs are introduced into the chromosome by expressing the I-SceI endonuclease (Fig. 5c and Additional file 3: Fig. S3C, E, F), and, if DSBs repaired, GFP is then expressed and subsequently quantified by flow cytometry. As shown, overexpression of OTUD4 resulted in a significant reduction of the percentage of GFP-positive cells in the DR-GFP U2OS but not that in the EJ5-U2OS reporter cells (Fig. 5d, Additional file 3: Fig. S3D), which indicated that OTUD4 inhibited DNA repair via HR signaling. Moreover, Rad51 staining confirmed that OTUD4 inhibited HR in NSCLC cells as indicated by less Rad51 foci in OTUD4 overexpressing cells after IR treatment (Fig. 5e, f).

## Discussion

A previous study reported that OTUD4 homozygous mutation was found in patients with ataxia and hypogonadotropic hypogonadism. Knockdown of *otud4* in zebrafish embryos induced defects in the eye, optic tectum, and cerebellum [22]. Up to date, this is the only report about deregulated OTUD4 in a pathological condition. Here, we report for the first time that deregulated OTUD4 associate with NSCLC. In this study, we found that OTUD4 was significantly downregulated in NSCLC cell lines and tumor tissues compared with normal controls (Fig. 1a–f). Analysis form Kaplan–Meier Plotter (http://kmplot.com) proves that the expression of OTUD4 positively correlates with the prognosis of NSCLC patients. Patients with lower OTUD4 expression show significantly shorter time of OS, FPS and PPS (Fig. 1g–i). These results indicate a tumor-suppressing role of OTUD4 the NSCLC.

OTUD4 has been reported to play multiple roles in DNA damage repair. Abigail Lubin and colleagues identified OTUD4 as a binding partner of XPC and modulating the ubiquitination of XPC [11]. XPC is an important positive regulator of NER [23, 24], thus they proposed that OTUD4 involved in NER. However, because ubiquitination of XPC had been proved both positively and negatively regulating NER [25–27], which might result from different type chain linkages of ubiquitination at different lysine residues, the exact role of OTUD4 in NER is not clear. By systematically analyzing, Yu Zhao et al. demonstrated that the OTUD4 could complex with USP7-USP9X. They proved that the OTUD4-USP7-USP9X complex was required for alkylation damage resistance and repair via promoting stability of ALKBH3, a demethylases for alkylation damage repair [12]. In our study, we find that OTUD4 could radiosensitize NSCLC cells by inhibiting the HR DNA repair signaling (Figs. 3 and 5), which broadened the role of OTUD4 in DNA damage repair.

OTUD4 was originally identified as a K48-specific deubiquitinase [28]. Very recently, Nima Mosammaparast et al. [29] proved that OTUD4 could switch to a K63-specific deubiquitinase upon phosphorylated near its catalytic domain. Numerous evidence have proved that ubiquitinase and deubiquitinase play important roles in DNA damage repair signaling transduction [30, 31]. According to a previous report, knockdown of OTUD4 increased the ubiquitination of XPC, which suggests the deubiquitinase activity of OTUD4 might be essential for NER [11]. Here, we show that OTUD4 inhibits HR repair (Fig. 5d, e). Yet, whether the deubiquitinase activity of OTUD4 involves in HR repair and what the exact mechanism is unexplored. Because K63 polyubiquitination plays pivotal roles in HR repair [32], we propose a hypothesis that OTUD4 might be phosphorylated by ATM and thus function as a K63-specific deubiquitinase to inhibit DSBs HR repair. Indeed, a SQ-rich region (aa334-aa458), which is characterized as the motif phosphorylated by ATM [33–35], is present in OTUD4 (data not shown). Nevertheless, Nima Mosammaparast et al. demonstrated that OTUD4 promotes alkylation damage repair by promoting stability of ALKBH3, which is independent on the deubiquitinase activity of OTUD4, but instead depends on the deubiquitinase activity of its interactors, USP7 and USP9X. Thus, OTUD4 might also function as an adapter and modulates HR repair signaling via other interactors. However, more investigations are needed to validate and uncover the underlying mechanisms.

Overall, in this study, we show that OTUD4 is silenced by promoter methylation and its downregulation correlates with poor prognosis in NSCLC. Overexpression of OTUD4 impairs DSBs HR repair, enhances cell cycle arrest and increases cell death induced that by IR. Our results suggest a tumor-suppressing function of OTUD4 and prove OTUD4 to be a potential target for radiosensitizing NSCLC.

## Conclusions

In conclusion, our data suggested that OTUD4 was downregulated in tissues and cells of NSCLC resulted from promoter hypermethylation. Downregulated OTUD4 correlated with poor prognosis of NSCLC patients. Ectopic overexpression of OTUD4 radiosensitized NSCLC cells via inhibiting HR repair of DSBs induced by IR, which further led to impaired clonogenic formation, enhanced cell apoptosis and cell cycle arrest. Collectively, this study showed that OTUD4 was a potential novel therapeutic target against NSCLC.

## Additional files

**Additional file 1: Fig. S1.** Overexpression of OTUD4 increases radiosensitivity of PC-9 cells. (A) Western blotting and real-time PCR validating overexpression of OTUD4 in PC-9. (B) Representative images of cell clonogenic formation. (C) Statistical quantification of cell clonogenic formation efficiency. α-Tubulin served as loading control. Error bars represent SD from 3 independent experiments. *, p<0.05.

**Additional file 2: Fig. S2.** Effects of OTUD4 on cell cycle and apoptosis in NSCLC cells. (A) Representative pictures of cell cycle distribution of indicated cells without IR. (B and C) Representative images (B) and statistical quantification (C) of NSCLC cells without IR. Error bars represent SD from 3 independent experiments. NS, not significant.

**Additional file 3: Fig. S3.**OTUD4 inhibits DNA damage repair. (A and B) Representative pictures (A) and quantification (B) of γ-H2AX foci in vector and OTUD4 overexpressed cells treated with IR (6Gy) and allowed recovering for indicated time. (C and D) Diagram (C) and homology repair efficiency (D) determined by FACS of EJ5-U2OS cells transfected with indicated plasmid. (E and F) Western blotting analysis of the expression of OTUD4 and HA-I-SceI in DR-GFP-U2OS (E) and EJ5-U2OS (F) Cells. Error bars represent SD from 3 independent experiments. *, p<0.05.

## Abbreviations

NSCLC: non-small cell lung cancer
LUAD: lung adenocarcinoma
LUSC: lung squamous cell carcinoma
IR: ionizing radiation
OTUD4: OTU deubiquitinase 4
OS: overall survival
PFS: progression free survival
PPS: post progression survival
DSBs: DNA double strand breaks
HR: homology recombination
GSEA: gene set enrichment analysis
