# Supplementary figures and images for: OTU deubiquitinase 4 is silenced and radiosensitizes non-small cell lung cancer cells via inhibiting DNA repair
Source: Cancer Cell Int. 2019 Apr 15;19:99. doi: 10.1186/s12935-019-0816-z (PMC6466656; doi:10.1186/s12935-019-0816-z)

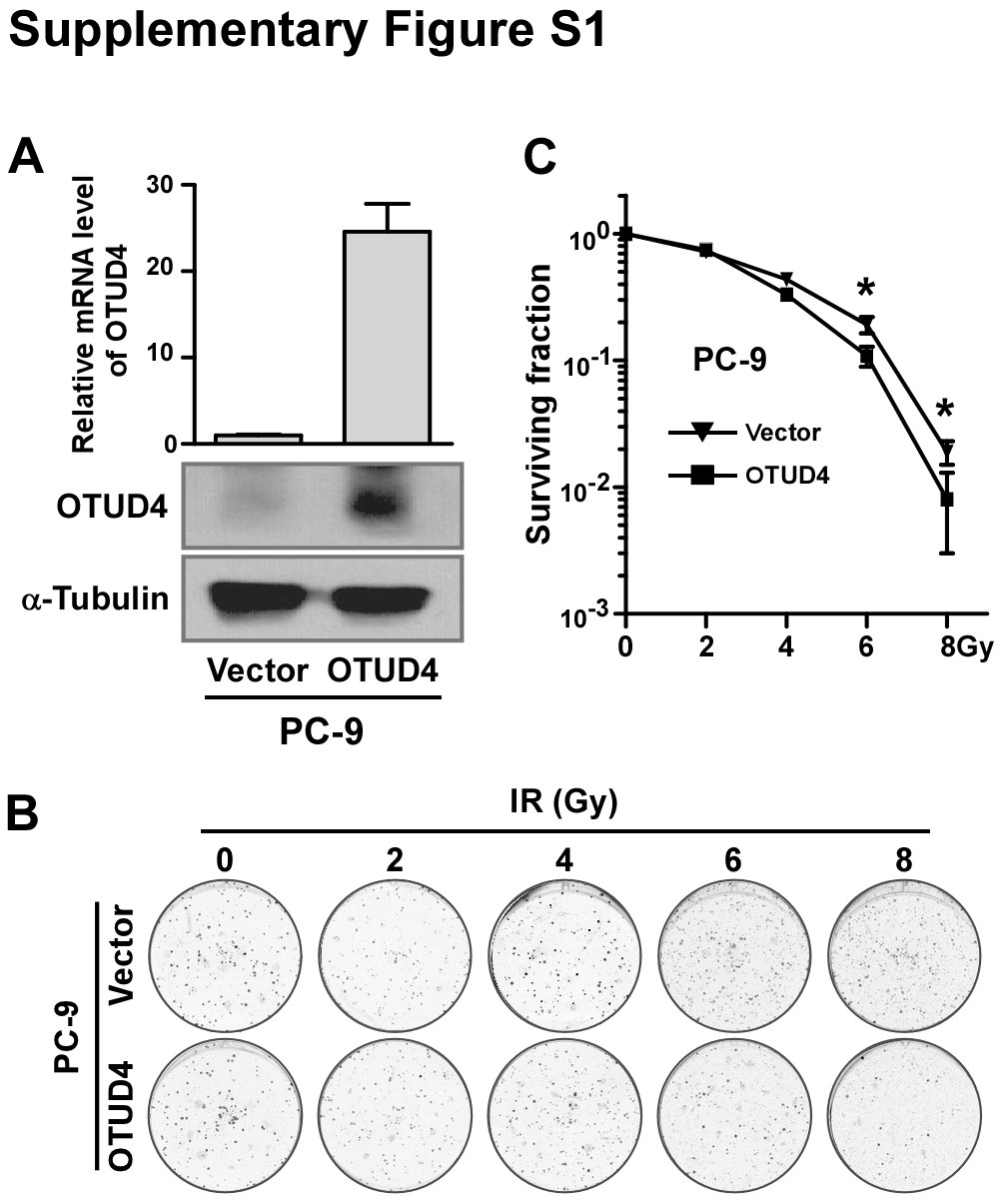

Supplement: Supplementary file 1 — Additional file 1: Fig. S1. Overexpression of OTUD4 increases radiosensitivity of PC-9 cells. (A) Western blotting and real-time PCR validating overexpression of OTUD4 in PC-9. (B) Representative images of cell clonogenic formation. (C) Statistical quantification of cell clonogenic formation efficiency. α-Tubulin served as loading control. Error bars represent SD from 3 independent experiments. *, p<0.05. [file 12935_2019_816_MOESM1_ESM.tif]

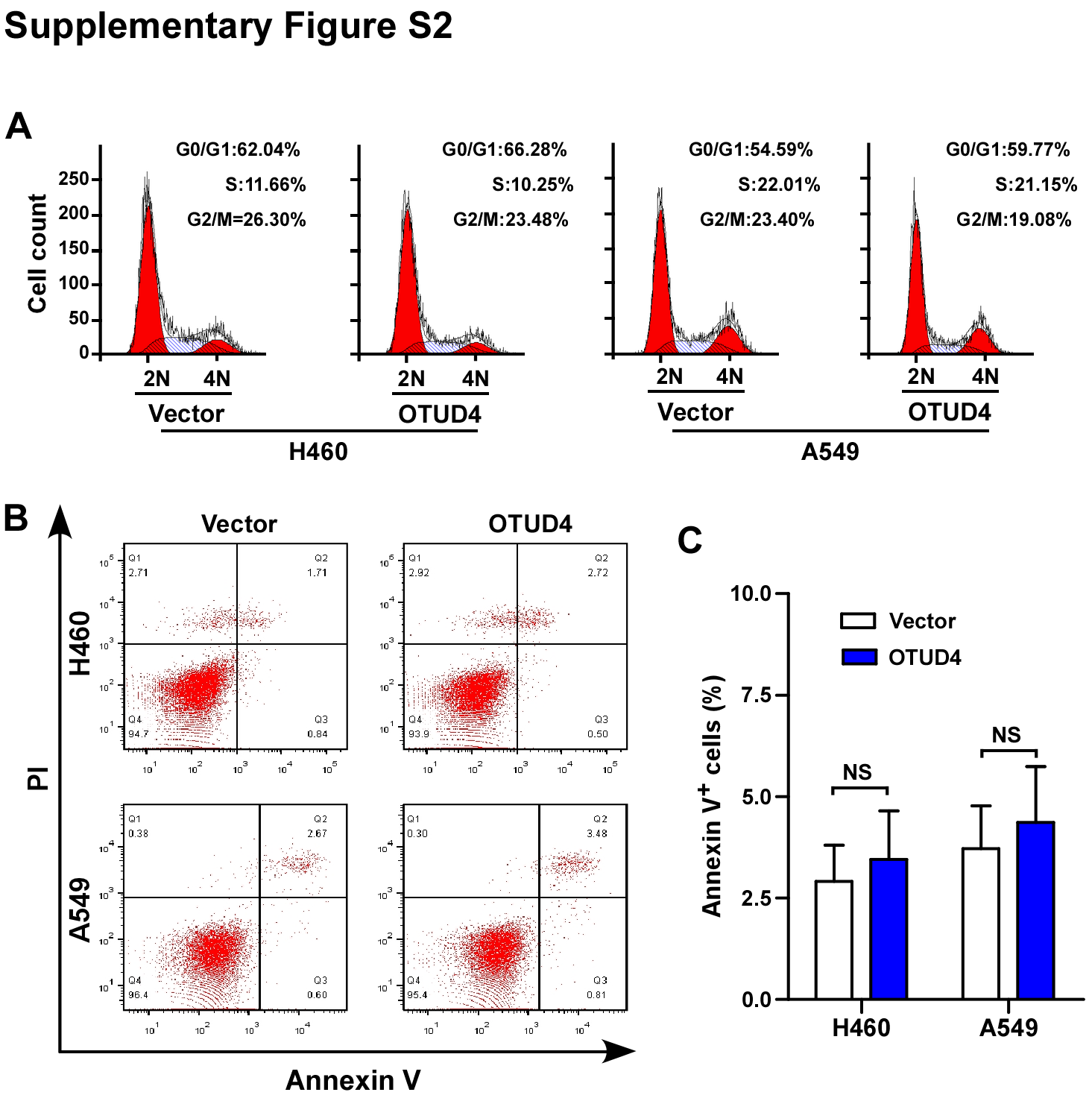

Supplement: Supplementary file 2 — Additional file 2: Fig. S2. Effects of OTUD4 on cell cycle and apoptosis in NSCLC cells. (A) Representative pictures of cell cycle distribution of indicated cells without IR. (B and C) Representative images (B) and statistical quantification (C) of NSCLC cells without IR. Error bars represent SD from 3 independent experiments. NS, not significant. [file 12935_2019_816_MOESM2_ESM.tif]

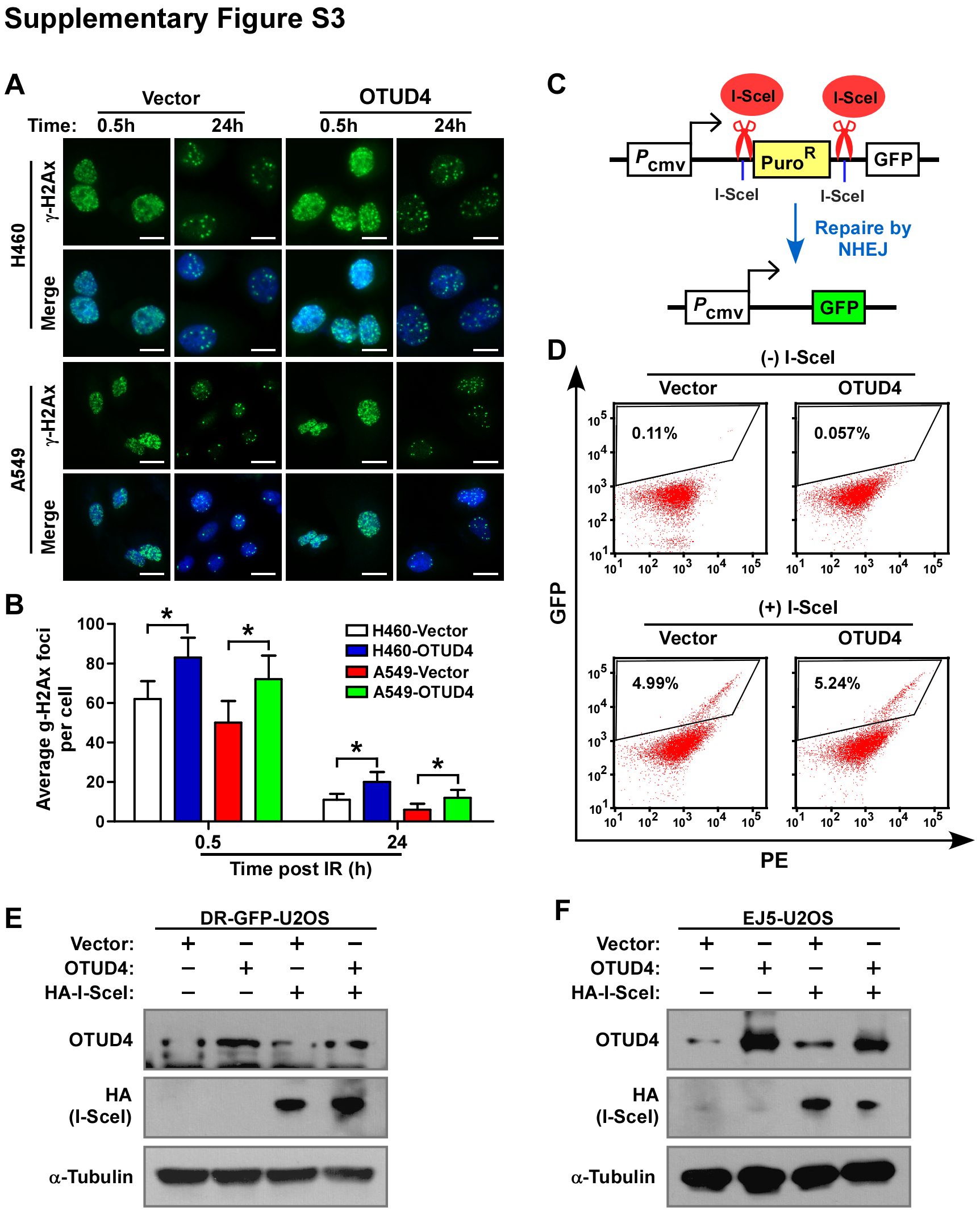

Supplement: Supplementary file 3 — Additional file 3: Fig. S3.OTUD4 inhibits DNA damage repair. (A and B) Representative pictures (A) and quantification (B) of γ-H2AX foci in vector and OTUD4 overexpressed cells treated with IR (6Gy) and allowed recovering for indicated time. (C and D) Diagram (C) and homology repair efficiency (D) determined by FACS of EJ5-U2OS cells transfected with indicated plasmid. (E and F) Western blotting analysis of the expression of OTUD4 and HA-I-SceI in DR-GFP-U2OS (E) and EJ5-U2OS (F) Cells. Error bars represent SD from 3 independent experiments. *, p<0.05. [file 12935_2019_816_MOESM3_ESM.tif]
